# Editorial: Mycorrhizal fungi and plants in terrestrial ecosystems, volume II

**DOI:** 10.3389/fpls.2023.1180884

**Published:** 2023-06-12

**Authors:** Mohamed Hijri, Amadou Bâ

**Affiliations:** ^1^ Institut de Recherche en Biologie Végétale (IRBV), Département de sciences biologiques, Université de Montréal, Montréal, QC, Canada; ^2^ African Genome Center, Mohammed VI Polytechnic University (UM6P), Ben Guerir, Morocco; ^3^ Laboratoire de Biologie et Physiologie Végétales, L’Unité de Formation des Sciences Exactes et Naturelles, Unité Mixte de Recherche 113 LSTM, Université des Antilles, Pointe-à-Pitre, France

**Keywords:** mycorrhizal symbiosis, ericoid and arbuscular mycorrhiza, plant-microbe interaction, mycorrhiza associated bacteria, fungi

In 1885, Frank reported a mutualistic symbiotic relationship between a fungus and its host in which both organisms nutritionally relied on each other (Frank and Trappe, 2005). He observed that mycorrhizal associations were widespread across a variety of habitats and soils, contrary to the general thinking of the nineteen centuries. This suggested that mycorrhizae play a substantial role in ecosystem diversity, functioning and stability. Arbuscular mycorrhizal (AM) associations are the most widespread among all types of mycorrhiza, estimated to involve more than 80% of all plant species. These associations are characterized by the formation of intercellular and intracellular hyphae, as well as specialized branching hyphae which create two distinct structures: coils in Arum-type and arbuscules in Paris-type arbuscular mycorrhizae, in the root epidermis and cortical cells. In some species, vesicles or swollen hyphal structures may also develop, both intra- and extraradical hyphae. AM fungal spores of certain genera are found on both intraradical and extraradical hyphae, and auxiliary cells are sometimes produced in the extraradical mycelium of some species. All of these fungi belong to the phylum Glomeromycota. AM symbiosis is found in a variety of plants, from liverworts and ferns to conifers and angiosperms. However, there are some angiosperms that do not form AM symbiosis. Among these non-host species members of Brassicaceae are believed to be a non-host for arbuscular mycorrhizal fungi (AMF), because they produce isothiocyanates that are involved in inhibiting arbuscular mycorrhizal symbiosis (Sharma et al., 2023). This view has been challenged by a study of Floc’h et al. in canola (*Brassica napus L.*), an important commodity crop. The authors implemented a lengthy experiment in three sites of the Canadian Prairies to determine the presence of AMF in the canola rhizosphere and bulk soil *via* metabarcoding on 18S rDNA targeting AMF. Fungal network analysis was then employed to assess the co-occurences between the AMF, fungi, and bacteria in the canola rhizosphere and bulk soil. The authors showed that the communities of AMF remain in the soil even when canola is cultivated as the sole crop for a decade. Furthermore, AM fungal taxa were identified in the close vicinity of the plant, which is not even a host for these fungi. They proposed that this phenomenon can be explained by the two possibilities: i) AMF may colonize the epidermis and outer cell layers of canola roots without forming a functional symbiotic relationship. This superficial colonization of canola roots may be enough for AMF to experience some growth and produce spores, thereby helping to maintain their presence. The same can be said for the root cortex of *Arabidopsis thaliana* (Cosme et al., 2018); ii) Fungi and Bacteria co-occuring with the mycelia of AMF could benefit colonization of canola roots by AMF (Floc’h et al., Basiru et al., 2023). Moreover, root endophyte bacteria can be recruited by AMF upon entering the root interior and may have beneficial effects on the associated plants where they can also augment mycorrhizal root colonization. Nevertheless, distinct genera were distinguished in the network analysis of AM fungal cohorts from the canola rhizosphere and bulk soil of the Canadian Prairies. The high frequency of Vicinamibacterales in the cohorts of AMF from these environments implies that bacteria may aid AMF in adapting to hostile conditions, or even function as AM fungal hosts.

A study of plant-plant interactions and coexistence was conducted to determine how phosphorus, nitrogen, and carbon resources were distributed between con- and heterospecific plant species in the presence or absence of AMF (Faghihinia and Jansa). The authors setup an experiment on two grass species belonging to the same genus and having contrasting response to mycorrhizal symbiosis *Panicum bisulcatum* (C_3_ and mycorrhizal-dependent) and *P. maximum* (C_4_, less mycorrhizal-dependent), either inoculated or not with *Rhizophagus irregularis*, were grown in mono- and mixed-cultures. They used radio- and stable isotopes to determine that in mono-systems, *P. maximum* gained more mycorrhizal phosphorus uptake benefits than *P. bisulcatum*. However, in the mixed culture, the *R. irregularis* appeared to transfer nutrients to *P. bisulcatum* more than *P. maximum*. Additionally, a higher ^13^C allocation to AMF by *P. bisulcatum* was observed in mixed- compared to the mono-systems, which likely improved the competitiveness of *P. bisulcatum* in mixed stands. These findings suggested that the presence of AMF influences the competitiveness of the two congeneric species of *Panicum* genus.

Complex interactions between plants, AMF and herbivores have an impact on plant functional traits, including reproduction. To explore the effects of AM fungal inoculation and herbivory on functional traits of the cultivated strawberry (*Fragaria x ananassa*), Whyle et al. conducted a field-based, potted-plant factorial experiment. They examined the influence of inoculation with AM fungal spores and herbivory on phenotypic plasticity in three different strawberry cultivars. Their findings revealed that genotype is a major factor in determining how herbivory and mycorrhizae affect strawberry functional trait expression. Furthermore, AM fungal inoculation was able to counter the negative impacts of herbivory on plant reproductive success. However, genotype will still determine the plant response. Therefore, the potential of AMF, and likely other beneficial microbes, for improving crop productivity depends on genotype and cultivar selection.

In another study, Thiao et al. aimed to investigate the interrelationship between trees, herbaceous plants on the ground, soil characteristics, and AM fungal communities. *Vachellia seyal* and *Prosopis chilensis* and their associated herb layers were the focus of the investigation. Samples of the soil beneath the trees and outside the canopies were subjected to physicochemical and microbial characterization. Additionally, living roots of trees and dominant herbs were randomly collected and tested for AM fungal colonization. The results indicated that the presence of trees improves herb richness and diversity. Furthermore, the soil mycorrhizal inoculum potentials were higher beneath *V. seyal* than *P. chilensis*, and decreased significantly with increasing distance from the trees.

Morvan et al. investigated the impacts of four varying levels of thermal pruning on wild blueberry performance, weeds, diseases, and the fungal and bacterial communities of the rhizosphere. A field trial was conducted with a randomized block design and agronomical variables were measured over a two-year period. MiSeq metabarcoding was used to analyze the diversity and composition of the bacterial and fungal communities. The results revealed that yield, ripeness of fruit, and several other agronomical variables were not significantly affected by the burning treatments. The only parameter that changed significantly was soil phosphorus, but only for a month after thermal pruning. Furthermore, bacterial and fungal communities did not demonstrate any significant variations between burning treatments. The dominant fungal species were ericoid mycorrhizal fungi, while the main bacterial species were Acidobacteriales, Isosphaerales, Frankiales, and Rhizobiales. Burning at high intensities briefly reduced septoria leaf spot in the season after thermal pruning. Altogether, the findings of this study show that thermal pruning has only a short-term effect on the wild blueberry ecosystem.

## Author contributions

MH wrote the text; AB commented and edited the text. All authors contributed to the article and approved the submitted version.
